# Mental health problems associated with female genital mutilation

**DOI:** 10.1192/pb.bp.114.047944

**Published:** 2015-12

**Authors:** Jeroen Knipscheer, Erick Vloeberghs, Anke van der Kwaak, Maria van den Muijsenbergh

**Affiliations:** 1 Arq Psychotrauma Expert Group, Diemen/Oegstgeest, and Department of Clinical and Health Psychology, Utrecht University, The Netherlands; 2 Pharos, Centre of Expertise on Health Disparities, Utrecht, The Netherlands; 3 Royal Tropical Institute and University of Amsterdam, The Netherlands; 4 Radboud University Medical Centre, Nijmegen, The Netherlands

## Abstract

**Aims and method**

To study the mental health status of 66 genitally mutilated immigrant women originating from Africa (i.e. Somalia, Sudan, Eritrea and Sierra Leone). Scores on standardised questionnaires (Harvard Trauma Questionnaire-30, Hopkins Symptom Checklist-25, COPE-Easy, Lowlands Acculturation Scale) and demographic and psychosocial correlates were analysed.

**Results**

A third of the respondents reported scores above the cut-off for affective or anxiety disorders; scores indicative for post-traumatic stress disorder were presented by 17.5% of women. Type of circumcision (infibulation), recollection of the event (a vivid memory), coping style (avoidance, in particular substance misuse) and employment status (lack of income) were significantly associated with psychopathology.

**Clinical implications**

A considerable minority group, characterised by infibulated women who have a vivid memory of the circumcision and cope with their symptoms in an avoidant way, reports to experience severe consequences of genital circumcision. In terms of public healthcare, interventions should target these groups as a priority.

Female genital mutilation (FGM) ‘comprises all procedures involving the partial or total removal of the external female genitalia or other injury to the female genital organs whether for cultural, religious or other non-therapeutic reasons’.^1^ The procedure is performed within a wide range of mainly African ethnic groups. Appropriate estimations are that between 100 and 140 million women and girls have undergone the practice.^2^ FGM is defined as a violation of human rights, and in many countries it has been outlawed. Nonetheless, FGM is still performed in a large part of Africa, particularly in Eastern regions. Currently, FGM is also performed in small migrant girls resident in European countries.^3^ The impact of FGM on social and psychological health is underresearched^4-9^ and knowledge of the psychosocial consequences in a migration context is even scarcer.^10-12^ Moreover, reports vary from high prevalence of trauma-related and other psychiatric symptoms such as anxiety and depression,^4,13^ to no mental health problems at all^14^ or even positive feelings.^15,16^

The aims of this study were to explore the relationship between FGM and psychopathology in circumcised women originating from an African country who have migrated to The Netherlands. Research questions were:

1. What kind of mental health problems are reported by circumcised immigrant women?
2. Which factors are associated with the presented psychopathology?

Based on the literature,^4-12^ we hypothesised potential predictors of psychopathology which could be distinguished between demographic factors (e.g. country of birth, years of residence in The Netherlands, educational level), circumcision-related factors (type of circumcision, age at the time of circumcision, vividness of recollection of the circumcision), coping mechanisms and acculturation demands.

## Method

### Design

In a cross-sectional research design, a survey among a sample of circumcised immigrant women in The Netherlands was conducted. Topics were measured in a quantitative way by means of culturally sensitive standardised questionnaires (the complete study design is described in Vloeberghs *et al*^17^).

### Procedure

The estimated size of the target population (women who have undergone FGM living in The Netherlands) is about 26 000 women.^3^ We involved representatives of this population actively in the research process, at the level of both data acquisition and data interpretation. Participants were recruited by means of snowball sampling, a sampling method that is being used to study hard-to-reach populations (see, for example, de Jong & van Ommeren^18^).

The first stage of snowball sampling involves selecting individuals using referrals by insiders within the target population. These individuals were asked to list others with identical characteristics. From this list at least one person was randomly selected and approached for an interview. The interviewee was then asked to list others and the same procedure was repeated several times. Attempts were made to maximise sociodemographic diversity. Approximately half to a third of the solicited women (depending on the interviewer and her network) agreed to participate. It was not possible to compare those who chose to participate in the interview with those who declined.

The interviews were conducted by seven interviewers (all females coming from the participants' countries of origin). We consulted various key persons in the different communities during the development of the measures and the interpretation of the results (e.g. during ‘experts meetings’). Interrater reliability across the interviews was enhanced by means of a protocol that guided the interview process and a training concerning the way in which the interviews should be conducted, how to handle possible risks of (re-) traumatisation and ethical aspects concerning the process of personal data collection.

The respondents were informed about the aim of the study by means of an information sheet. The interviewers – who were awarded financial compensation – were instructed to read the information sheet to illiterate respondents. It was emphasised that they had to guarantee anonymity and that participation of the respondents was voluntary. Participants were reassured about confidentiality and told that they were not obliged to answer questions that they did not wish to answer. They were given the telephone number of a family doctor attached to the project (M. van den M.) whom they could contact in case of problems or questions concerning their well-being.

The interviews mainly took place at the respondents' homes. In some cases they were conducted at community centres or in public places, depending on the preference of the participant. The interviews were completed within an 18-month period in 2008/2009. The duration of the interviews varied from 40 to 180 min (mean duration about 90 min). The answers were recorded verbatim and later transcribed by the researchers (J.K. and E.V.).

### Sample characteristics

A group of 66 women participated in the study (Table 1). The participants' ages ranged from 18 to 69 years, averaging 35.5 years (s.d. = 10.5); 43% were married, 79% had children (mean 1.78, s.d. = 1.6). The group included 36 (54%) heavily circumcised (infibulated) women, mainly from Sudan and Somalia. The other women had undergone less invasive forms of circumcision: 9 women got an excision (type 2), whereas 21 had undergone type 1 circumcision.^19^ The age at which the participants were circumcised ranged from 8 months to 16 years (mean 6.4, s.d. = 4.1).

**Table 1:** Descriptive statistics of demographic variables of the FGM sample (*n* = 66)

Variable	
Age, years: mean (s.d.) range	35.5 (10.5) 18–69

Age at circumcision, years: mean (s.d.) range	6.4 (4.1) 0.8–16

Years in The Netherlands, mean (s.d.) range	10.9 (6.3) 2–29

Number of children, mean (s.d.) range	1.78 (1.6) 0–8

Country of birth, *n* (%)	
Somalia	18 (27)
Sierra Leone	12 (18)
Sudan	18 (27)
Eritrea	12 (18)
Ethiopia	6 (9)

Type of mutilation,^a^ *n* (%)	
Type I clitoridectomy	21 (32)
Type II excision	9 (14)
Type III infibulation	35 (54)

Marital status, *n* (%)	
Alone (single, widow, divorced)	33 (57)
Married with family	25 (43)

Education, *n* (%)	
Low (⩽6 years)	9 (16)
Middle (6–12 years)	24 (43)
High (⩾12 years)	23 (41)

Source of income, *n* (%)	
Job, education fee or social benefit	37 (66)
No income	19 (34)

FGM, female genital mutilation.

a. According to World Health Organization classification.^19^

### Instruments

The survey consisted of four questionnaires including the Harvard Trauma Questionnaire (HTQ-30),^20^ a 30-item transculturally validated screening instrument for post-traumatic stress disorder (PTSD) symptomatology (Cronbach's α = 0.96 in the current sample); the Hopkins Symptom Checklist (HSCL-25),^21^ which measures anxiety (10 items) and depression symptoms (15 items) and has proven to be useful as a screening instrument in several cross-cultural studies and patient studies^22-25^ (Cronbach's α = 0.96); the COPE-Easy,^26^ which measures different coping styles by means of 32 items grouped under three theoretical head dimensions: (a) actively problem-directed coping, (b) support-seeking coping and (c) avoidance behaviour; the internal consistency of the subscales of COPE-Easy in this sample was satisfactory (Cronbach's α varying between 0.67 for avoidance behaviour and 0.91 for active problem-directed coping); and the Lowlands Acculturation Scale (LAS),^27^ which assesses the level of cultural adaptation with 20 items and distinguishes between a global orientation towards the past (and land of origin) as opposed to the orientation towards the future (and country of current residence) in terms of integration skills and culture-bound traditions (Cronbach's α = 0.63). All instruments were translated into languages spoken by the participants, applying a back-translation procedure. A preliminary version of the questionnaires was pilot-tested with ten women and both content and format were revised on the basis of results.

### Statistical analysis

Hierarchical regression analyses were used to test whether demographic factors (country of birth, age, years of residence in The Netherlands, marital status, educational level, source of income and number of children), circumcision-related factors (type of circumcision, age at the time of circumcision, vividness of recollection of the circumcision), coping strategies (COPE-Easy subscales) and acculturation demands (LAS subscales) predicted symptom severity of PTSD (HTQ-30 total score), anxiety and depression (HSCL-25 total score).

## Results

More than a third of the participants (*n* = 24, 36%) scored above the cut-off level for indicators of psychopathology: a fifth of the total sample (*n* = 13, 20%) met the criteria for PTSD (mean HTQ-30 score >2.5), a third met the criteria for depression (*n* = 22, 33%), nearly a third met the criteria for an anxiety disorder (*n* = 20, 30%; mean HSCL-25 score >1.75) and a sixth (*n* = 11, 18%) scored above the cut-off level for all three psychopathology indicators. Almost two-thirds of all participants (*n* = 42, 64%) did not report scores above the cut-off on indicators for PTSD, anxiety or depression.

Type of circumcision, country of origin, source of income, vividness of recollection and coping style were significant factors in a multivariate context concerning mental health symptoms. Infibulation, a ‘vivid recollection’ and a substance-misuse coping style were associated with enhanced PTSD scores, whereas originating from Somalia was associated with decreased post-traumatic symptoms (*R*^2^ = 0.67, *F*_(4.38)_ = 22.04, *P*<0.0001; Table 2). Associated with higher anxiety and depression scores were infibulation, substance misuse, avoidance coping and lack of income; however, women originating from Somalia reported less anxiety and depression (*R*^2^ = 0.59, *F*_(5.39)_ = 13.68, *P*<0.0001; Table 3).

**Table 2:** Summary of hierarchical multiple regression analysis on HTQ-30 total score (*n* = 66)

Variable	Beta	95% CI low	95% CI high
1 Memory	−0.648***	−0.644	−0.296

2 Memory	−0.522***	−0.536	−0.222
Coping substance misuse	0.421***	0.062	0.194

3 Memory	−0.545***	−0.542	−0.248
Coping substance misuse	0.422***	0.067	0.190
Somalia	−0.255**	−0.739	−0.101

4 Memory	−0.478***	−0.489	−0.204
Coping substance misuse	0.335***	0.041	0.163
Somalia	−0.358***	−0.915	−0.263
Infibulation	0.285**	0.076	0.625

HTQ, Harvard Trauma Questionnaire.

* *P*<0.05

** *P*<0.01

*** *P*<0.001.

**Table 3:** Summary of hierarchical multiple regression analysis on HSCL total score (*n* = 66)

Variable	Beta	95% CI low	95% CI high
1 Coping substance misuse	0.656***	0.140	0.293

2 Coping substance misuse	0.551***	0.101	0.263
Coping avoidance	0.254*	0.002	0.094
3 Coping substance misuse	0.546***	0.102	0.258
Coping avoidance	0.289*	0.009	0.099
Somalia	−0.219*	− 0.781	0.000

4 Coping substance misuse	0.467***	0.078	0.230
Coping avoidance	0.207	−0.005	0.083
Somalia	−0.322**	−0.970	−0.183
Infibulation	0.316*	0.090	0.737

5 Coping substance misuse	0.454***	0.076	0.223
Coping avoidance	0.224*	0.000	0.084
Somalia	−0.330**	−0.969	−0.210
Infibulation	0.284*	0.059	0.687
No income	0.199*	0.001	0.547

HSCL, Hopkins Symptom Checklist.

* *P*<0.05

** *P*<0.01

*** *P*<0.001.

## Discussion

FGM is associated with a wide range of long-term health and psychological problems (e.g. Andro *et al*^28^). Circumcised immigrant women in this study are likely to report emotional disturbances that relate to FGM, with about a sixth reporting scores above the threshold for PTSD and a third reporting severe levels of depression or anxiety. Specific factors, associated partly with FGM and partly with current life stressors, influence the severity of psychopathology. In particular, infibulated immigrant women who have a vivid recollection of the circumcision, who do not have a paid job, and who cope with their problems mainly in an avoidant way (substance misuse), may form a group at risk of severe psychological problems.

The majority of the sample reported mental health problems but did not meet criteria indicative of psychopathology. These relatively low percentages could be due to the common fact that the majority of the survivors of traumatic events are able to recover without developing mental health problems. The underreporting of symptoms could also be owing to different perceptions (not the circumcision but other stressors would be responsible for the current complaints/symptoms) or taboo (being ashamed to talk about the problems, feeling a sense of stigma). In addition, the reluctance to speak out can be related to the fact that thinking or talking about the mutilation may cause the pain experienced at the time of the ritual to reappear; chronic pain and traumatisation can have a mutually reinforcing effect.^29^ Nonetheless, it may also be true that the majority of the women experience no substantial traumatic symptoms as a consequence of FGM (see Lockhat,^12^ who showed that women who were circumcised according to the mild sunna variant (type 4) did not report PTSD-related problems).

The finding that infibulation and a vivid recollection may enhance symptomatology may not come as a surprise. Still, some findings do puzzle us, such as the finding that Somali background appears to have a protective effect.

Speaking openly about the trauma may only be beneficial when this is appropriate within a culture of recognition of the trauma and its psychological consequences. Somali respondents may have more problems communicating about FGM in their culture. Another explanation may be that Somali women assess the event less negatively than women from other countries. According to the Lockhat model,^12^ a negative judgement is predictive of PTSD development. In fact, some Somali women refer to the Islamic teachings saying that sunna is not prohibited. To them sunna has a normative and positive connotation within Islam, whereas, for instance, Sudanese respondents reject the Somali reinterpretation (Idjtihad) of what is being said in the Holy Writings.^11^

Another finding concerns the relationship between symptoms, avoidant coping and substance misuse. Avoidance plays a pivotal role in PTSD symptom maintenance.^30^ Accordingly, it is not surprising that avoidant coping is associated with PTSD symptom severity. Participants may develop substance misuse problems in an attempt to manage distress associated with the effects of FGM and related stress symptoms, or to numb themselves from the remembrance or experience of intense emotions. In our sample this coping style seems to exacerbate the problems in those experiencing anxiety and depression.

Our study has a unique character. The active participation of the target population and the way we obtained data provide sufficient ground for answering the research questions validly. For HTQ and HSCL excellent cross-cultural psychometric results have been reported. Some caution, however, is warranted in interpreting the results. Using a cross-sectional design, we can only employ a relatively passive approach to making causal inferences based on the findings – we may only speak about potential predictors. Moreover, the small group size, which is an inherent consequence of studying such a precarious topic, presents an important limitation as do the composition characteristics of the group (i.e. the high level of education and low marital state).

Incorporated in the clinical treatment of circumcised immigrant women should be providing them with coping mechanisms to help them come to terms with their experiences. Using therapy to equip women with instrumental skills to help them cope in day-to-day life and promote social integration to avoid social isolation (e.g. by enrolling the women in education/training) is also a good starting point for improving mental health (see also Summerfield^31^). In addition, when treating women who experienced FGM one must be able to discern the various types of FGM, be knowledgeable about the related symptoms and the effects these may have on the woman, and have awareness regarding the taboo surrounding the practice. Finally, mental healthcare providers should be attentive to the fact that FGM is but one of a range of possible traumatising experiences the patient may have been subjected to. They should not only focus on FGM but check whether there are other factors, such as social or financial circumstances, that may be causing the symptoms presented by the patient.

On the basis of the empirical findings presented, our study shows that FGM is associated with psychological health problems for a substantial group of immigrant women. However, a considerable number of women are capable of coping with most impediments and may regard the ritual as ‘normal’ and therefore not sickening. Our data thus underline the diversity in interpreting the events and the level of remembrance as crucial for experiencing psychopathology. For future studies, it is important to elucidate the contextual factors that influence decisions about service utilisation. Prevention and clinical efforts should focus on the individual within its context and should be aware of potential hesitation among some women to seek psychological help. Clinicians are encouraged not to pathologise the consequences of FGM but to focus on the urgent psychological, social and psychosexual needs identified among a significant number of circumcised women.
